# Pre-Hispanic fishing practices in interfluvial Amazonia: Zooarchaeological evidence from managed landscapes on the Llanos de Mojos savanna

**DOI:** 10.1371/journal.pone.0214638

**Published:** 2019-05-15

**Authors:** Gabriela Prestes-Carneiro, Philippe Béarez, Myrtle Pearl Shock, Heiko Prümers, Carla Jaimes Betancourt

**Affiliations:** 1 Anthropology and Archaeology Program, Institute of Social Sciences, Federal University of Western Para, Santarém, Pará, Brazil; 2 Archéozoologie, archéobotanique: sociétés, pratiques et environnements (UMR 7209), CNRS, Muséum national d’histoire naturelle, Paris, France; 3 Kommission für Archäologie Aussereuropäischer Kulturen, Deutsches Archäologisches Institut, Bonn, Germany; 4 Institut XI Abteilung für Altamerikanistik und Ethnologie, Universität Bonn, Bonn, Germany; Institut Català de Paleoecologia Humana i Evolució Social (IPHES), SPAIN

## Abstract

Recent evidence suggests the existence of Pre-Hispanic fisheries in savanna areas of the Amazon basin. How these fisheries may have functioned is still poorly known. Although many studies have drawn attention to how Pre-Hispanic inhabitants of these savannas managed to deal with excess water, little attention has been paid to understanding how large and permanent populations were sustained during long periods of drought. In the Llanos de Mojos, one of the largest savannas in South America, the landscape is greatly affected by the impacts of annual, seasonal flooding and inundations, alternating with a dry period that can last 4–6 months. The fishing practices in this area were studied on the basis of analysis of more than 17,000 fish remains recovered at Loma Salvatierra, a monumental mound located in an interfluvial area 50 km from the Mamoré River and occupied between 500 and 1400 AD. In Loma Salvatierra, a network of circular walled ponds connected to a system of canals has been identified, raising questions about a possible use of these structures for fishing. The exceptional conservation of the bone material has enabled precise taxonomic identification of more than 35 taxa, the richest fish spectrum thus far documented in the Mojos region. The dominant fish, swamp-eels (*Synbranchus* spp.), armored catfishes (*Hoplosternum* spp.), lungfish (*Lepidosiren paradoxa*), and tiger-fish (*Hoplias malabaricus*) are characteristic of shallow and stagnant waters. Our work documents the first zooarchaeological evidence of a dryland, interfluvial fishing system in the Bolivian Amazon that incorporates distinct species and fishing practices, demonstrating that these regions contain year round resources. Research is taking its first steps toward understanding landscape modifications, fish environments, and specific cultural technologies employed on this and other lowland neotropical savannas that differ from those for fishing in open waters and rivers.

## Introduction

The analysis of bone remains can be a window to reconstruct part of the functioning of managed landscapes such as pre-Hispanic fisheries. Advances in Amazonian archaeology have demonstrated that the major rivers of the Amazon basin and most of its tributaries, such as the Napo, Madeira, and Tapajós Rivers, were densely populated during the first millennium AD [1–3]. Aquatic resources (fish and reptiles), extremely abundant in such areas, constituted the main components of Pre-Hispanic economies [4,5]. In interfluvial areas, however, the access to aquatic resources is less evident, availability of fish is conditioned by strong seasonal constraints and the acquisition of animal protein constitutes, therefore, an issue still under investigation [6–9].

The issue of subsistence of societies living along major rivers versus societies settled in interfluvial areas constitutes a debate held by many scholars since the early 1950´s. Steward (1948) [10] suggested that “(…) what is thought of as a typical Tropical Forest culture or selvan culture–(…) flowed along the coast and up the main waterways (…) leaving the hinterland tribes on a more primitive level” and argued that when forests were replaced by savannas, inhabitants would abandon agriculture, turning their economy towards wild food. In the same way, interested in Pre-Hispanic economies of the Central Amazon, Lathrap (1968) [6] proposed that the lack of protein resources in interfluvial areas limited the development of forest tribes. During the last 15 years, studies carried out in savanna areas of the southwestern border of Amazonia, the Llanos de Mojos region, have called into question many of the statements concerning social organization of societies that occupied interfluvial areas. Research has documented large and elaborate earthworks, constructed throughout the first millennium AD, attesting to the development of large and sedentary societies in the region. In areas more than 50 km from the closest major river (Mamoré), networks of ditches, causeways and canals associated with raised fields suggest that these human groups managed to adapt their economy to savanna environmental settings [11–13].

Characterized by extremely flat terrain, the Llanos de Mojos are greatly affected by large oscillations of the water level along the year. Nordenskiöld (2003 [1911], our translation) [14], one of the first ethnographers traveling in the region, quoted “*Mojos is a strange land*, *a land of contrasts*. *During the dry season it is difficult to get a drop of water on that same plain*, *and you can often see the land far around devastated by fire*. *During the rainy season*, *the neighbors visit each other in a canoe*. *At the time of the drought you can see these canoes in the middle of the plain*, *where there is not the slightest humidity*, *and those who are unfamiliar with the conditions could easily ask*: *what on earth do these people want to do in the dry land with canoes*? *Mojos is really a strange country; there you swim with the ox-team and go by canoe in the countryside”*. From December to March, more than 50 percent of the territory is flooded. In this regard, many scholars have attempted to investigate how Pre-Hispanic societies were able to manage the twin constraints of drought and flooding. Archaeological records, such as the presence of cultivated plants (maize, manioc, and sweet potatoes) associated with raised fields in many Llanos de Mojos sites constitute substantial evidence that ancient economies transformed flooded savannas into agricultural landscapes [14–17]. Raised fields are one of the most impressive earthworks of the savanna, however there are many types of landscape transformations such as the monumental earthen mounds, locally called “lomas”. The variation in type of construction, their implantation in the environment and relationship with settlements has led to the division into different archaeological zones [18,19].

From April to November, waters recede, and rainfall is very sparse, during an extreme dry period lasting four to five months of the year. The lack of water during the dry period was first addressed by Denevan (1966) [15]. The author pointed to the role of artificial ditches as water-retaining systems during the periods of drought. The issue was later studied by Erickson (2000) [8], working in Baures (about 180 km northeast of the study case area). The area is located in an interfluvial savanna area located about 50 km from two rivers, San Joaquin and San Martin. The author described zig-zag ridges of raised earth that crossed the savanna. These structures were interpreted to be vestiges of fish weirs and Erickson (2000) claimed that the system could have provided drinkable water and fish during the dry season. More recently, McKey *et al*. (2016) [20] presented a present-day Zambian fishery showing landscape patterns very similar to those described in Baures. Although most researchers agree that such structures might have been (directly or indirectly) related to fishing activity and to the management of water storage [21], no systematic excavations have been carried out in the fish weirs zone and no zooarchaeological research has been conducted in the area so far.

The importance of fishing to savanna groups of the Llanos de Mojos was also documented at the end of the 17^th^ century by the first Jesuit missionaries to the region. An anonymous author who lived in the Llanos de Mojos in 1754 wrote *“The most ordinary livelihood of these Indians is fish*, *that is plentiful in these rivers and lakes”* [22]. Although such written sources give precious knowledge about past dietary habits in the Llanos de Mojos, their scarcity makes it difficult to evaluate the role of fishing in the economy during the transition pre-contact/contact period. Preliminary zooarchaeological research carried out at the Loma Salvatierra site by Driesch *et al*. (2012) [23] demonstrated that cervids such as red brocket (*Mazama americana*) and gray brocket (*M*. *gouazoubira*), nutria (*Myocastor coypu*), agouti (*Dasyprocta variegata*), paca (*Agouti paca*), and the muscovy duck (*Cairina moschata*) were among the most represented taxa recovered at the site. Although fish corresponded to about 40% of the total number of identified remains during phases 3 and 4, the high diversity of fish species in the region and lack of extensive reference collections prevented accurate identification of most fish remains. Domesticated plants were recovered at Loma Salvatierra however the areas cultivated have not been identified as there are no raised fields in this zone.

Loma Salvatierra is a constructed earthen mound, continuously occupied from AD 500 to AD 1400, that extends over an area of 2 ha, from which a system of canals and ponds radiates. In this paper we present detailed taxonomic identification of 17,338 fish remains coming from different occupational periods of the site, represented by archaeological excavations across more than 100 m^2^. The sample enables the reconstitution of possible fishing environments and their distribution over the landscape, and a discussion of other South American fisheries. Considering this example, an archaeological site situated far from permanent water bodies, we aim to discuss how and by what means large populations of the Llanos de Mojos dealt with long periods of drought and lack of water.

### Environmental setting

The Llanos de Mojos is one of the largest tropical savannas in South America, extending over 200,000 km^2^, and is about eighty percent covered by low vegetation (grasslands and scattered short trees) interspersed with patches of forest galleries in the transition zone between the Amazonian equatorial rainforest and the semi-deciduous Chiquitano Dry Forest.

Four major rivers drain the Llanos de Mojos territory: Mamoré, which constitutes the central axis of the Llanos de Mojos, Madre de Dios, Beni, and Itenez/Guaporé, all tributaries of the Madeira River. On its right bank, the Mamoré has only one large tributary, the Ibare River [24]. Seasonality plays a major role in the landscape transformations of the Llanos de Mojos. Given the poor drainage of the soils associated with the flatness of the terrain, the Mamoré River and its tributaries cannot handle the volume of water, driving the flooding of the adjacent areas from October to April. The months of greatest precipitation, from December to February, result in subsequently higher flooding from March to April. Hamilton et al. (2004) observed between 1979 and 1987 that the maximum total flooded area was 78 000 km^2^, but it is known that the extent of inundations vary considerably by year [25].

The waters invading the plain are divided into two categories: the so-called “white-waters”, which are of Andean origin, highly mineralized and heavily loaded in suspended organic and inorganic matter and the “black-waters”, which are endogenous and stagnant waters formed as a result of local precipitations [24,26].

**Fig 1:**
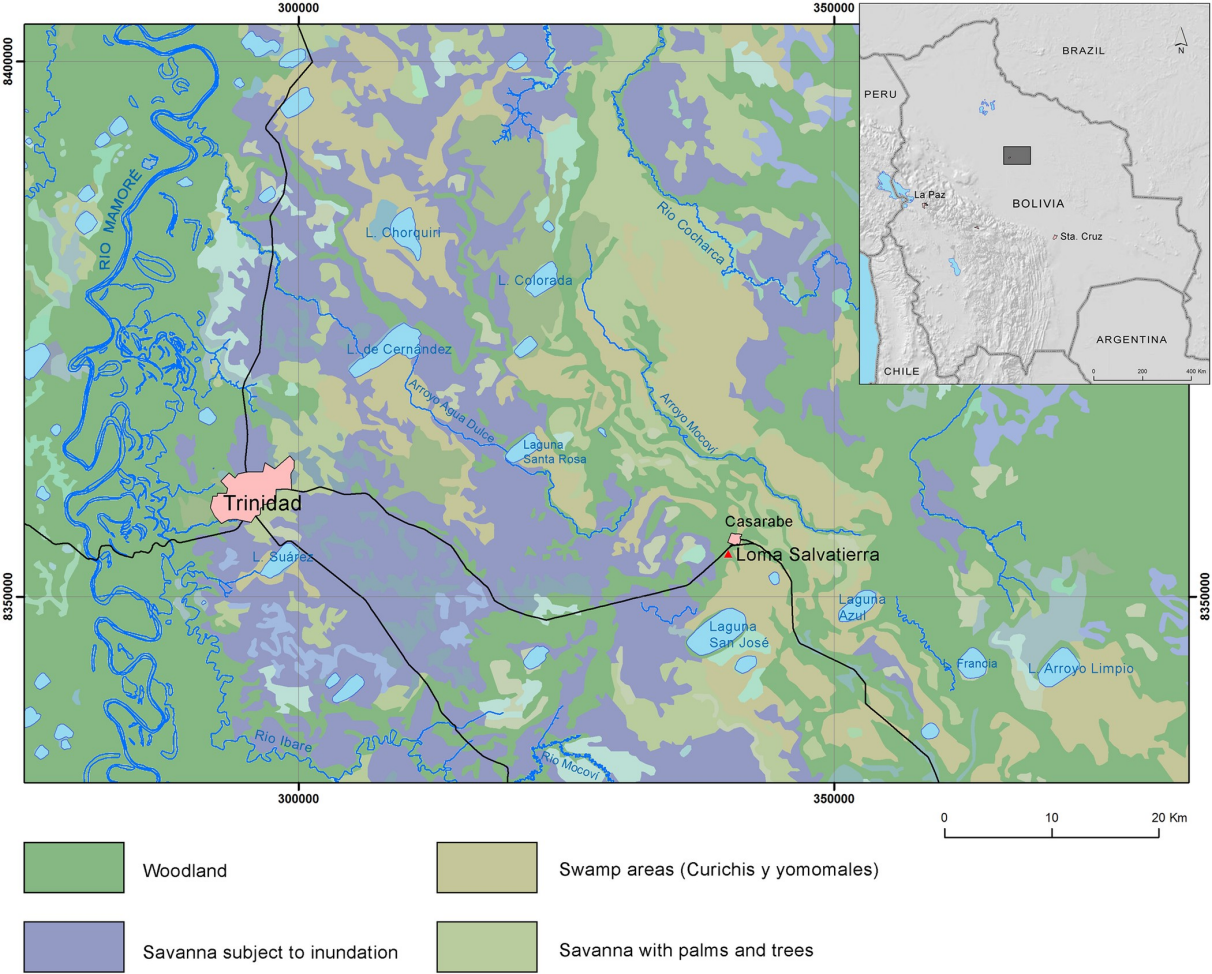
Geographical and hydrological situation of Loma Salvatierra. The terrain of the map is between 120 and 140 m above the sea level. Thick blue lines represent permanent rivers, thin blue lines indicate intermittent rivers. The original map has been redesigned by GPC and HP from the American Defense Mapping Agency (sheet 3941 of Series H632 maps—Bolivian topographic maps 1:100,000).

**Fig 2:**
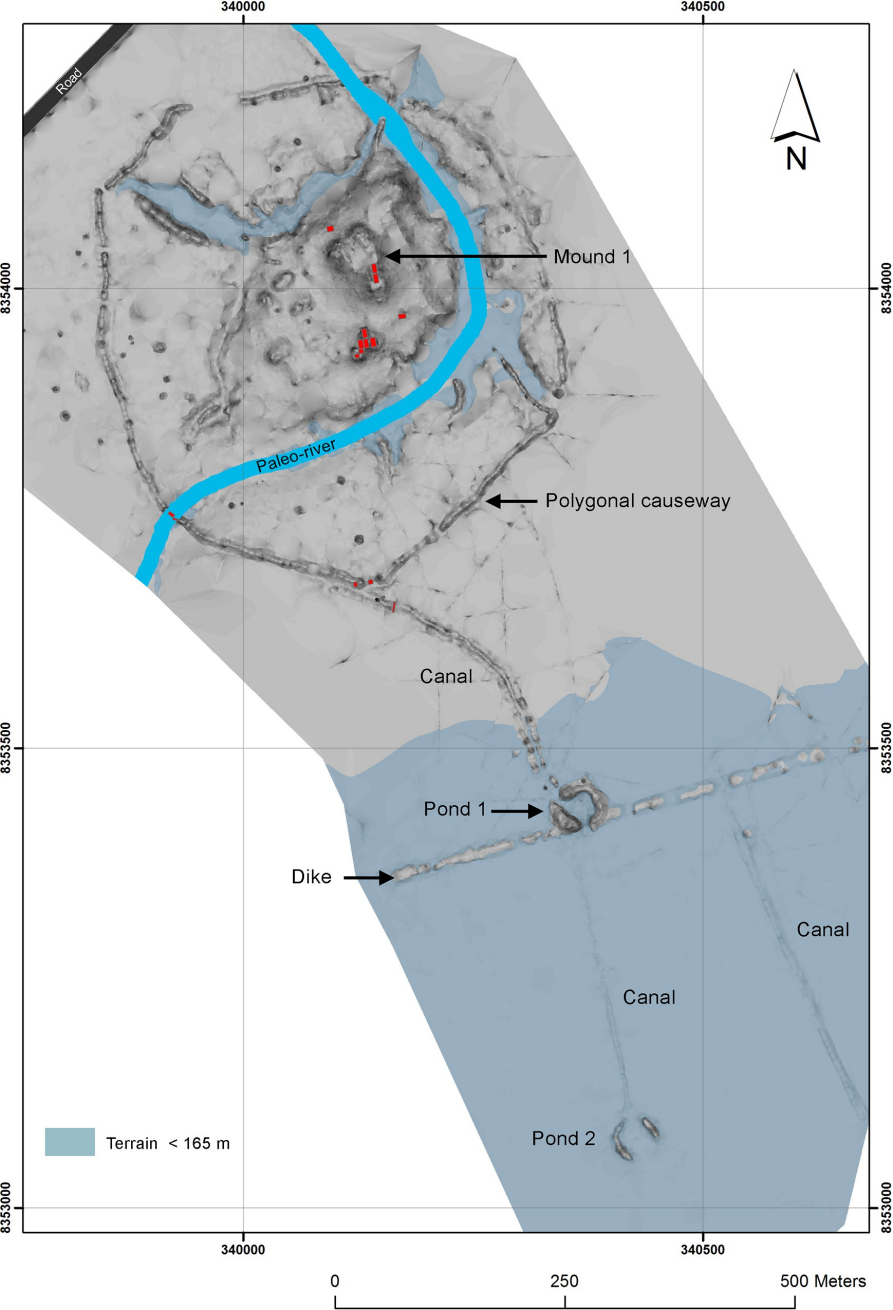
Artificial system of ponds and canals at Loma Salvatierra. Topographic map of Loma Salvatierra showing the network of canals linked to a pond system.

The studied area, the Loma Salvatierra zone (Fig 1), is located beyond the extent of the Mamoré’s annual flooding. Thus, inundations result from local precipitation that infiltrates the soil profile and accumulates more quickly than it can run off the plain (the mean annual precipitation varies from 1300 to 2000 mm across the Llanos de Mojos) [15,25,27,28]. As the water recedes, part of the water that covers the plains drains into the numerous streams and creeks that join the rivers while another part gets confined in shallow depressions across the plain (Figs 1 and 2). Such dynamics give rise to a mosaic of different stagnant and shallow-water environments that can be classified according to their permanent or temporary nature and to the proportion of surface occupied by vegetation. Lakes (or *lagunas*) are permanent all year round and generally not covered by vegetation, while pools (*pozas*) are seasonal and partially covered by floating vegetation. Swamps are almost entirely covered by the vegetation and generally dry up as water recedes. *Curiches* are old meander cutoffs that, despite the long periods of drought, can retain water all year. The community of plants that covers the body of such stagnant lentic waters is called “*Pantanos de Yomomos*”. *Cyperus* spp. (*junquillo*) and *Pontederia subovata* (*tarope*) are among the most common aquatic plants present in these lakes and ponds [24,29].

Animal and plant communities can vary considerably between water bodies as a function of their connectivity or isolation. Lakes located within about 10 km of the Mamoré River may be invaded by flooding white-waters that engender the dispersal of aquatic organisms and may experience large oscillations of the water level [28,30]. The lakes situated in the middle of the savanna (Laguna San José, Laguna Azul, and others) (see Fig 1), also called “savanna lakes”, however, are subjected to an independent hydrologic cycle dependent on local rainfalls. These lakes are very shallow (between 1.5 and 3 meters) and their water level varies less than in lakes connected to river flooding [30]

The hydraulic configuration and seasonal dynamics of the Llanos de Mojos greatly affected the cultures that settled in the region in pre-Hispanic times [15]. The environmental preconditions of the Llanos de Mojos constituted an important factor of emergence of the so-called “Monumental Mounds Culture” during the last part of the Holocene [11,31]. These societies inhabited the southeastern part of the Llanos de Mojos, an area that covers about 4,500 km^2^ east of Trinidad at the right margin of the floodplain of the Mamoré River [18,32].

### Loma Salvatierra site

The Loma Salvatierra site is one of the best studied sites of the “Monumental Mounds Region” and is situated about 50 km east of Trinidad and about 1 km from Casarabe village. The site is one among a set of more than 100 anthropogenic earthen mounds. Archaeological research assumes that most of these sites were constructed between AD 500 and AD 1400 [33]. The mounds were often constructed in forested areas (often near ancient levees of paleo-river channels) and their dimensions are around 20 m height and 6 ha in extent. They are connected to forest islands and other mounds through networks of canals and causeways. About 80% of these mounds are located more than 2 km from a water body [11].

Loma Salvatierra, an elevated earthen platform that extends over 2 ha, is about 6 km from the nearest permanent source of water, the Laguna Azul. The top of the platform is formed by three mounds, disposed in a “U” shape, the highest point reaching 7 meters above the surrounding plain. Loma Salvarierra is surrounded by a polygonal causeway that encircles an area of 21 ha, possibly delimiting the settlement zone. Detailed mapping of the archaeological site revealed that it is located at the margin of a paleo-river channel [18]. Since the polygonal causeway cuts the paleo-river channel, it is known that the creek was inactive before the settlement was occupied.

The area around Loma Salvatierra is relatively flat and the present-day vegetation is predominantly grass-lands with herbaceous aquatic macrophytes. The higher ground remnant of paleo-fluvial activity has patches of evergreen forest. While it is beyond the flooding of the Mamoré River, during the rainy season 5 to 10 cm of water (coming from local precipitation and increased soil saturation) can cover the terrain [34]. After the rainy season, the terrain gradually dries, surface water is absent, and saturated soils are restricted to the depressions of the paleo-river (see Fig 1).

**Fig 3:**
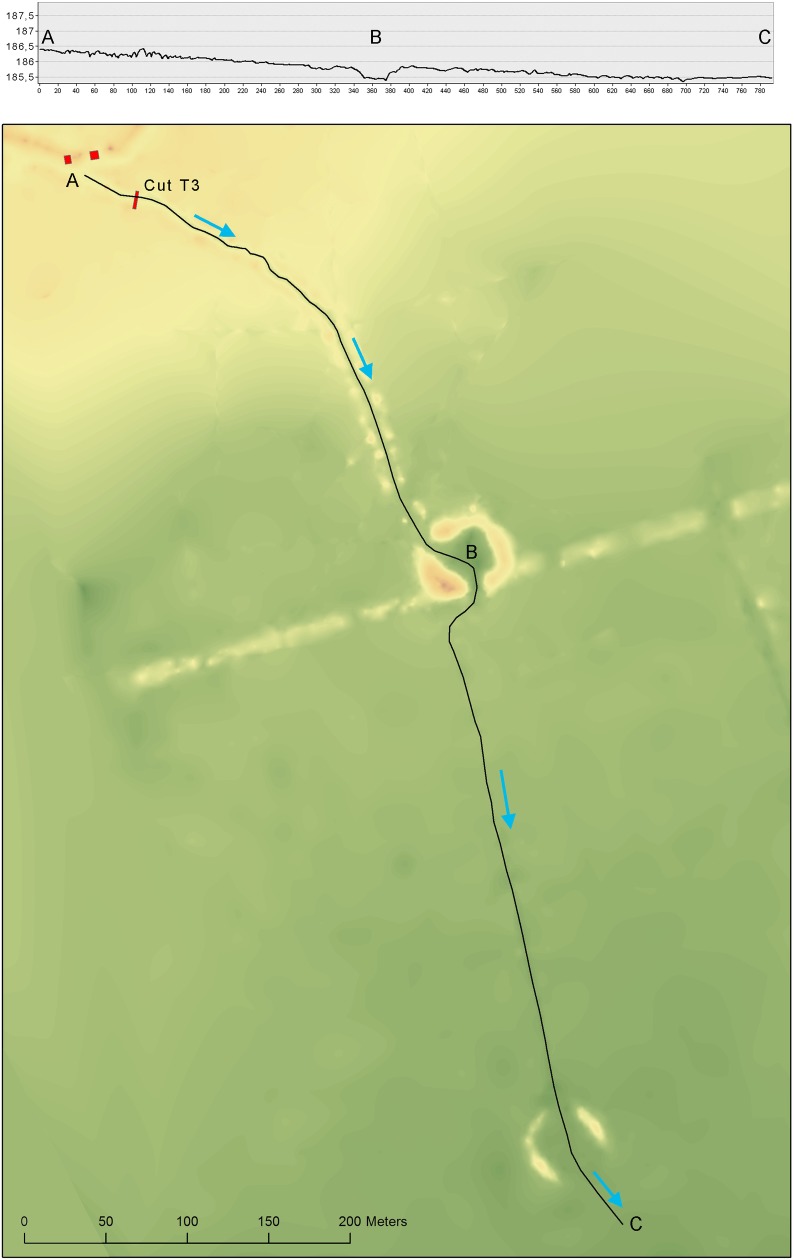
Detail of the topographical survey of circular structures.

**Fig 4:**
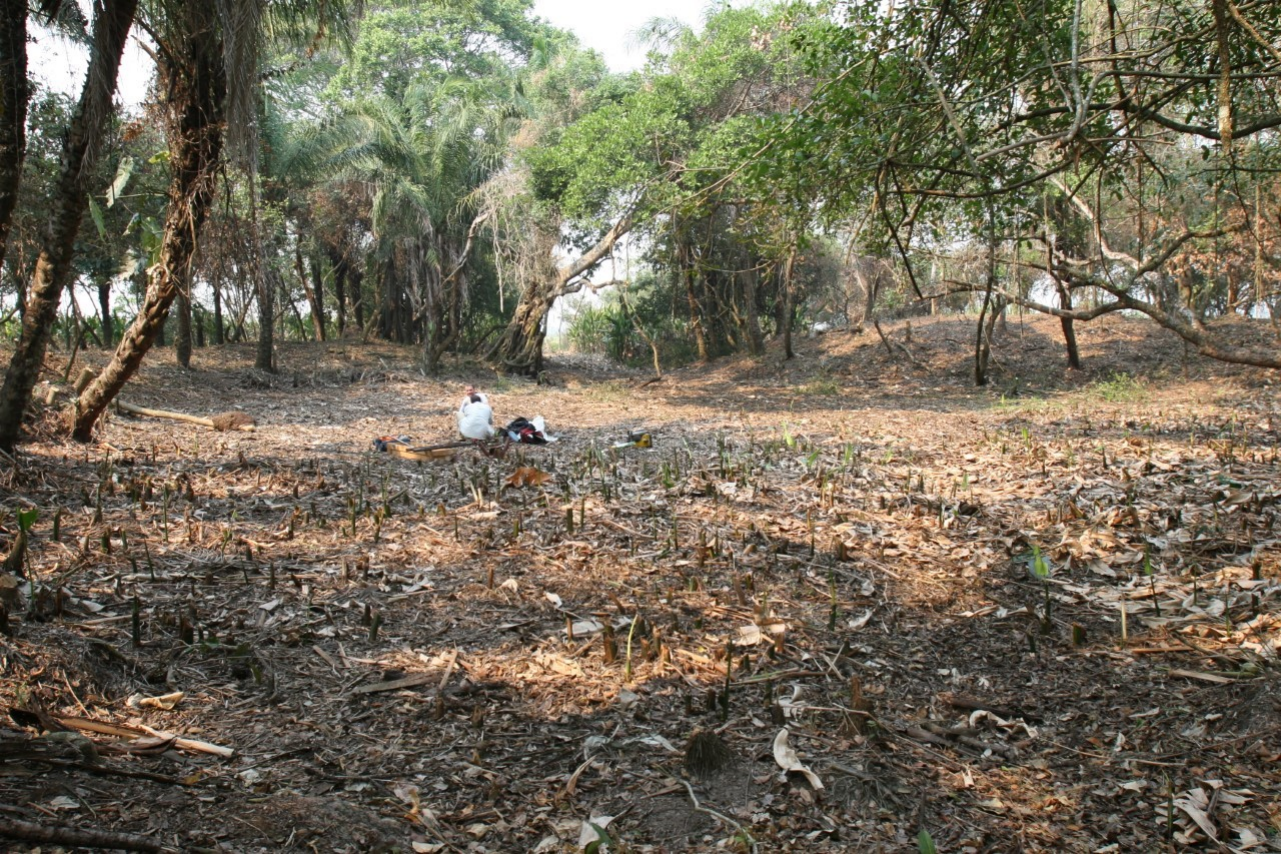
Interior of circular pond with canal exit visible in the center of the far margin.

To the southwest of Loma Salvatierra, a set of canals (about 300 m long) links the mound to an excavated pond, which is itself connected to a second set of canals that flow into another pond (Figs 2–4). This system likely acted like a funnel draining the rainwater towards ponds and canals during the period of receding waters [35].

A series of 34 radiocarbon dates indicate that the site was almost continually occupied between 500 cal AD and 1400 cal AD [33,36]. Although Loma Salvatierra ceramics present a homogeneous technological tradition, ceramic assemblages show particular temporal and regional traits, revealing that Llanos de Mojos was by no means a culturally homogeneous area. Morphological and decorative attributes of the ceramics recovered at the site allowed Jaimes Betancourt (2012) [33] to distinguish five occupation phases.

The study of macro-botanical remains revealed that a large range of plants were processed at Loma Salvatierra [16,37]. Maize was the most commonly cultivated plant, alongside peanut and squash. Starch grain analysis revealed the presence of manioc, chili pepper, yam and cotton. Despite the absence of evidence for raised fields in this region, it has been suggested that canals could have integrated a drainage network that allowed agriculture in flooded areas [32]. Furthermore it has been suggested that areas of the savanna between Loma Salvatierra and Lake San José (Fig 1) were cultivated without raised fields given the consistent presence of maize pollen in lake sediments corresponding chronologically with the occupation [38]. Other than fish, deer species (Mazama spp.), nutria (*Myocastor coypu)*, crab-eating fox (*Cerdocyon thous)*, six-banded armadillo *(Euphractus sexcinctus)*, Muscovy duck (*Cairina moschata*), lizards and caimans were among the most common vertebrate taxa recovered at the site [23].

## Materials and methods

Fish bones analyzed were sampled from 62 archaeological stratigraphic units from Excavation Units (EU) 2 and 4, the two best-studied archaeological contexts of Loma Salvatierra.

EU 2 measured 8m x 6m and was located on the main platform of the site (southwestern corner: UTM 20S 340169.35 m E, 8353966.86 m S). Excavations uncovered contexts belonging to the whole occupation sequence of the site and reached sterile soil. Findings related to food preparation, spinning, and manufacturing of bone and wooden tools indicate that the area of EU 2 was associated with domestic activities.

EU 4 was located on top of the central earthen mound (Mound 1) of the site and measured 10m x 5m (southwestern corner: UTM 20S 340140.82 m E, 8354016.53 m S). The abundance of nicely decorated sherds of fine ceramics [or non-utilitarian ceramics] has been taken as an indicator that festivities could have taken place in that area. Bowls of one meter depth, found in contexts of EU 4 belonging to the third occupation phase, were probably associated with preparation of fermented beverages (*chicha*) from maize or manioc. Excavations of EU 4 had to be stopped (for security reasons) at 4 meters depth. Thus, for this central part of the site only contexts of occupation phases 3, 4 and 5 have been documented [33].

Two different recovery methods were combined during the excavation of Loma Salvatierra: hand collecting and water sieving over 2 mm, 1 mm and 0.5 mm meshes. The majority of the identified material was hand-collected, although we also analyzed sieved material of at least 15 liters of sediment per layer (of both stratigraphic cuts), with the exception of layer 2 of EU 2, for which only 1.5 liters were sieved.

The osteological and taxonomic identifications of the specimens were undertaken with the aid of a comparative collection of about 120 specimens from the Amazon basin housed at the Muséum national d’histoire naturelle in Paris. Cranial and post-cranial remains were quantified using the Number of Identified Specimens (NISP) and the Minimum Number of Individuals (MNI). MNI was calculated on the basis of the best-represented anatomical element, often cranial elements (e.g. dentary, premaxilla).

In order to have a general idea of the size of archaeological specimens, we estimated the fish body length by comparing the archeological element with a reference collection specimen of the same taxon. Only for *Synbranchus marmoratus* did we apply an allometric body size estimation model [39]. The regression equation was elaborated using 68 modern specimens of *S*. *marmoratus* recovered in artificial ponds near Trinidad. The equation was subsequently applied to the archaeological assemblage.

## Results

Considering the usually rapid decomposition of organic matter in lowland tropical environments, the preservation of bone material was very good at Loma Salvatierra. Exceptionally, even a few scales were recovered, even though few could be identified under the family level. Bones from deeper layers were better preserved than those from upper layers, indicating that construction of the mound, comprised of large quantities of ceramics and clay soil, sealed and protected deeper layers from the effects of temperature variation and rainfall at the surface. Bones from EU 4 (stratigraphic units 429, 4018) were particularly well preserved compared to those from EU 2.

A total of 17,338 fish remains were analyzed, of which about 63% were identified to at least the order level. Thanks to the use of 2 mm, 1 mm and 0.5 mm mesh sieving, small-sized species such as 20 g characids or cichlids were described for the first time at the site. As expected, the larger bodied fish, swamp eels (Synbranchidae) and lung fish (Lepidosirenidae), were more abundant, or overrepresented, in the hand collected samples than water screened samples (respectively 74% and 39% of identified remains; S1 Table). Within each phase, the distribution of fish between the remaining orders was similar in samples from hand collection and water screening with the exception of smaller taxa that were better represented in water screened material. The use of an accurate reference collection also permitted the identification of a highly diverse spectrum (Table 1) that includes 35 genera grouped in 7 orders and 17 families. Dominant taxa will be commented upon below.

**Table 1:** Number of Chondrichthyes and Osteichthyes per phase recovered in EUs 2 and 4.

Taxon	Vernacular name (from [48])	Phase 1		Phase 2		Phase 3		Phase 4		Phase 5		Total NISP	Total MNI
		NISP	MNI	NISP	MNI	NISP	MNI	NISP	MNI	NISP	MNI		
**Myliobatiformes**	*Arraya*									1	1	1	1
**Characiformes**	* *							42	1	2	2	44	3
Anostomidae*	*boga*, *piau*	2	2					1	1			3	3
Curimatidae	*Sabalina*					1	1					1	1
Characidae	* *												
sp.1*	*Sardina*									1	1	1	1
sp.2*	*Sardina*							1	1			1	1
sp.3*	*Sardina*									1	1	1	1
Serrasalmidae UID	*Palometa*	2	1			2	2	22	3	1	1	27	7
*Mylossoma* sp.*	*Pacupeba*	2	1									2	1
*Serrasalmus* spp.*	*Piraña*					1	1	1	1	1	1	3	3
*Pristobrycon* sp.*	*Palometa*							2	1			2	1
*Pygocentrus nattereri*	*piraña roja*	1	1	1	1			7	5			9	7
Erythrinidae	* Tiger fish*	21	10	1	1	13	2	102	3	15	1	152	17
*Hoplias malabaricus*	*bentón*, *trahira*	53	10	6	4	113	18	423	21	13	4	608	57
*Hoplerythrinus* sp.*	*yeyu*, *yayu*	29	8			49	7	115	2	1	1	194	18
**Siluriformes UID**	* *	15	6	2	1	22	1	24	1	2		65	9
Auchenipteridae	*Torito*	2	2									2	2
Doradidae UID	* *	1				4	1	3	1			8	2
*Anadoras weddellii*	*Requereque*	4	2	13	3							17	5
*Ossancora punctata*	*Requereque*			1	1							1	1
*Oxydoras niger*	*tachacá*,*cuiu cuiu*							2	1			2	1
*Pterodoras granulosus*	*tachacá*,*itagivá*							2	1	1	1	3	2
Callichthyidae UID	* armored catfishes*	186	26	40	17	406	51	889	29	242	3	1763	126
*Hoplosternum littorale*	*buchere*, *tamoatá*	15	9			83	25	654	12	4	2	756	48
Megalechis picta*	*Simbao*					16	7	35	3	26	5	77	15
*Corydoras* sp.*	*Caracha*							72	37			72	37
Pimelodidae UID	* *	2	2					3	3			5	5
*Pimelodus* sp.	*chupa*, *bagre liso*			2	1	5	4					7	5
*Pseudoplatystoma* sp.	*Surubí*					3	3	3	2	3	2	9	7
Heptapteridae UID	* *			1		6	1					7	1
*Rhamdia* sp.*	*bagre*, *ñurundiá*			2	2	2	1					4	3
Loricariidae UID	* Zapato*	4	3	20	7	50	20	116	10	8	4	198	44
*Liposarchus* sp.	*caranxo*, *vieja*	3	3	16	11	6	6	44	6	2	1	71	27
Gymnotiformes*	*Cuchillo*							3	1			3	1
**Synbranchiformes**	* Swamp-eels*												-
*Synbranchus madeirae*	*Anguila*, *muçum*	8	5	19	7	55	22	22	17	7	3	111	54
*Synbranchus marmoratus*	*Anguila*, *muçum*	299	108	286	98	756	337	370	169	120	74	1831	786
*Synbranchus* sp.	* *	551	12	502	3	1103	29	1649	11	187	3	3992	58
**Cichliformes**	* *												-
Cichlidae UID	*Serepapa*	29	5			11	2	228	4	21		289	11
sp. 1*	* *									1	1	1	1
sp. 2*	* *									1	1	1	1
sp. 3*	* *									1	1	1	1
*Cichla monoculus*	*tucunaré*, *yacundá*					2	2	3	1			5	3
*Acaronia* sp.*	*acará*,*kupaka*					12	1					12	1
*Astronotus* sp.*	*serepapa real*					2	2	8	3			10	5
*Cichlasoma* sp.*	*Serepapa*	1	1					10	1	1	1	12	3
*Crenicichla* sp.*	*pez jabón*					3	2	8	2			11	4
**Perciformes**													
Sciaenidae	* *												
*Plagioscion* sp.	*pescada*, *corvina*					1	1					1	1
**Ceratodontiformes**	* *												-
*Lepidosiren paradoxa*	*pez pulmonado*, *lungfish*	39	15	31	15	218	97	298	65	74	30	660	222
**Total NISP**	* *	**1269**		**943**		**2945**		**5162**		**716**		**11056**	
Teleostei UID		1699		2		885		3557		139		6282	
**TOTAL**		**2968**	**232**	**945**	**172**	**3830**	**646**	**8719**	**419**	**855**	**145**	**17338**	**1614**

UID means unidentified remains.

Presence of * indicates taxa that were only recovered in sieved samples and absent in hand collected samples.

Here less attention is given to the chronological changes in the fish spectrum due to minimal data on environmental changes that would influence fish habitats. However, we note some general fluctuations in the predominant taxa. Swamp-eels (Synbranchidae) proportions exhibit a gradual decrease during phases 3 and 4 and rebound during phase 5, armored catfish (Callichthyidae) progressive increase from phases 1 to 4, followed by an abrupt decline in phase 5, and lungfish exhibit an increase in phase 5 (Fig 3). Fluctuations in the fish spectrum observed from phases 3 to 4 and 4 to 5 may be related to cultural and/or landscape transformations occurring around 1100 AD in Loma Salvatierra. This is suggested by evidence of reconfiguration of the site and material culture observed by Prümers *et al*. (2012) and Jaimes Betancourt (2016) [40,41].

**Fig 5:**
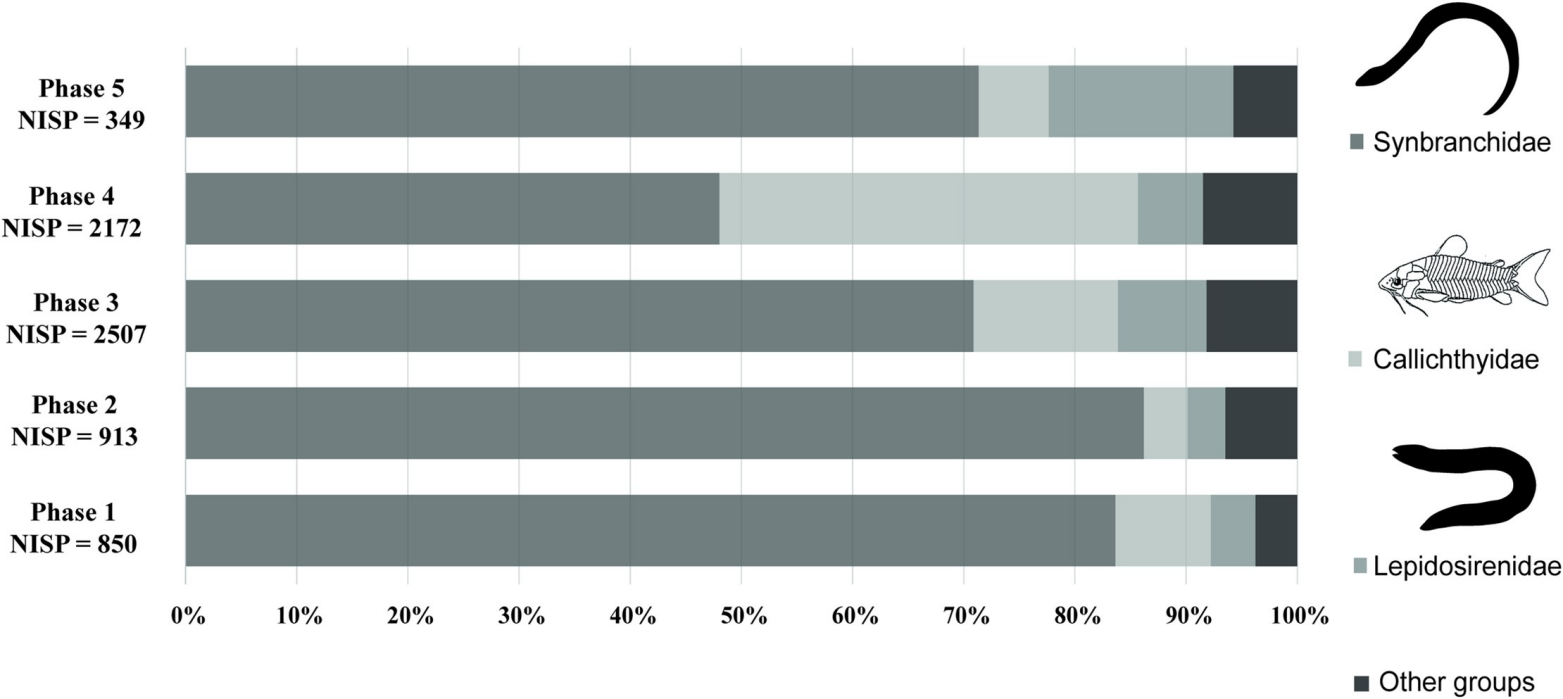
NISP distribution of the main families of hand-collected fish assemblages. Selection of only hand-collected remains was chosen so that material from two recovery methods were not mixed and so phase 2 was not misrepresented due to its low volume of water screened samples. The figure over-estimates the importance of larger taxa and larger individuals in the assemblage as detailed by S1 Table.

It is worth noting that there was a great difference between the spectra provided by hand-collected and sieved assemblages in species and numbers. In hand-collected assemblages, swamp eels (Synbranchidae) largely dominate the spectrum during all phases of occupation, reaching more than 80% of the assemblages of phase 2 (Fig 5). Armored catfishes or *buchere* (Callichthyidae), lungfish (*Lepidosiren paradoxa*) and Erythrinidae (tiger fish or *benton*) were also well represented, but their proportions fluctuated over time.

The sieved material is comprised of highly diverse small-sized fishes, including undetermined small sardines (Characidae), pirañas (Serrasalmidae) and *serepapas* (Cichlidae) (Fig 6.7–13). Among catfishes, a high diversity of species was observed ranging in size from small thorny fishes (*Ossancora punctata*, *Anadoras weddellii*) and barbeled catfishes (*Rhamdia* sp., *Pimelodus* sp.) to large-sized thorny fishes (*Oxydoras niger*, *Pterodoras* sp.) and tiger-striped catfishes (*Pseudoplatystoma* sp.) (Fig 6.14–19). Tiger-striped catfishes recovered at Loma Salvatierra had estimated sizes varying from 8 to 10 kg, and they must have represented an important resource in terms of protein intake.

**Fig 6:**
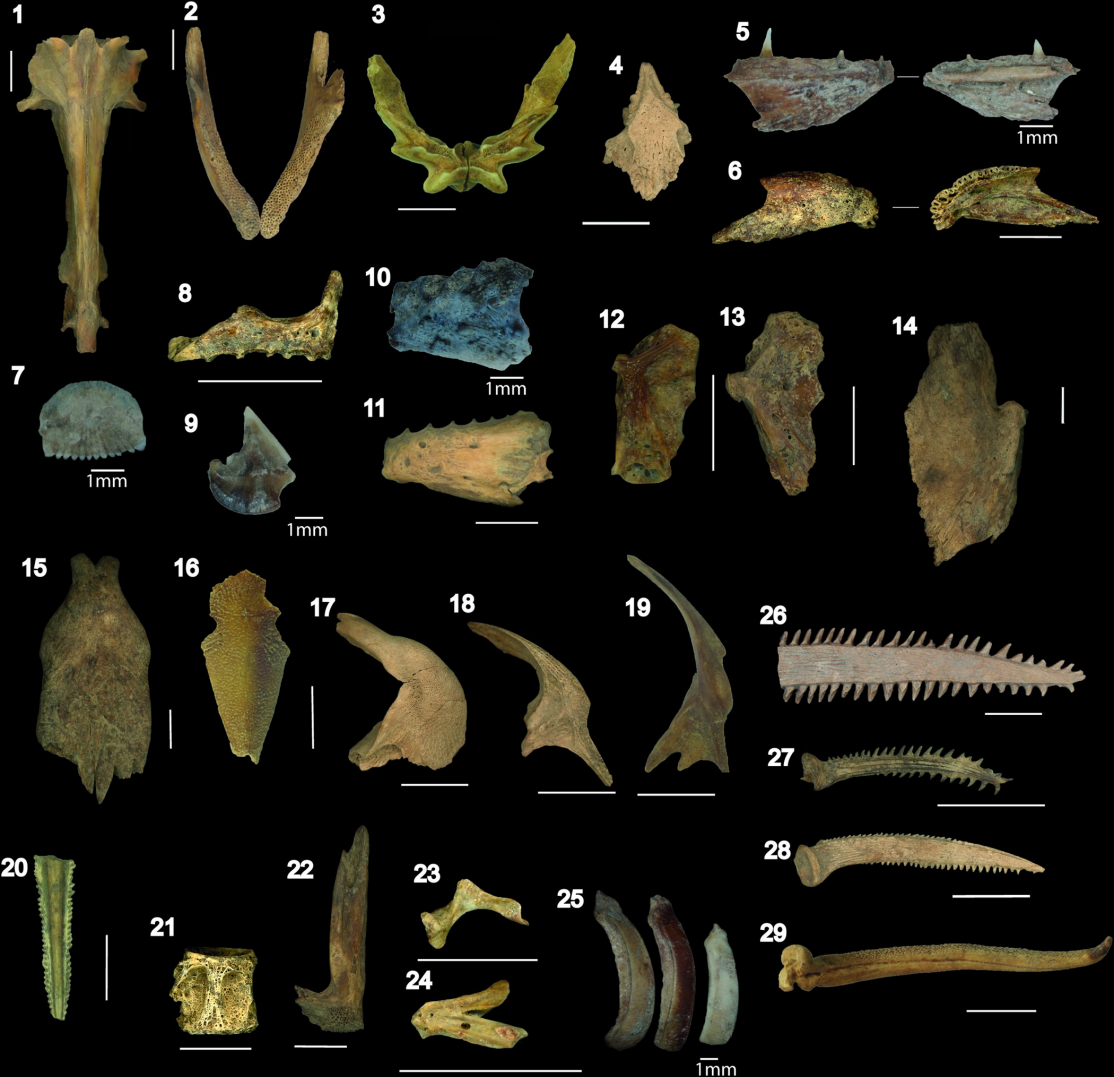
Fish remains from Loma Salvatierra. (1–2) *Synbranchus* sp. neurocranium and dentaries; (3) *Lepidosiren paradoxa* prearticular; (4–5) *Hoplias malabaricus*, ethmoid and dentary; (6) *Hoplerythrinus unitaeniatus* dentary; (7) Undetermined ctenoid scale; (8) cf. *Pristobrycon* sp. premaxilla; (9) Serrasalmid tooth; (10) cf. *Mylossoma* sp. dentary; (11) *Serrasalmus* sp. dentary; (12) Anostomidae hyomandibular; (13) Curimatidae hyomandibular; (14) *Pseudoplatystoma* sp. quadrate; (15) *Oxydoras niger* mesethmoid; (16) *Pimelodus blochii* supraoccipital; (17) Loricariidae cleithrum; (18) *Anadoras* sp. cleithrum; (19) *Rhamdia* sp. cleithrum; (20) Myliobatiformes caudal spine; (21) *Plagioscion* sp. vertebra; (22) *Cichla* sp. premaxilla; (23–24) *Cichlasoma* sp. maxilla and dentary; (25) Callicthyidae bony plates. Pectoral spines of (26) *Pterodoras granulosus* (27) *Anadoras* sp; (28) *Pimelodus* sp. and (29) *Hoplosternum* sp. Scale bar: 1 cm, except where 1 mm is noted.

Even though a large number of small-sized Callichthyidae were counted (about 25% of the NISP), if we consider their net weight, they correspond to only 1% of the total fish body mass, while swamp eels account for about 54% of the net weight of total fish remains and lungfish for 17%. This indicates that, in terms of biomass, small catfishes may not have had the same importance as larger fishes (biomass estimation).

Uncommon taxa include indeterminate sting-rays (Myliobatiformes), large catfishes (*Pseudoplatystoma* sp., *Pterodoras granulosus*), large cichlids (*Cichla* spp./tucunaré), sciaenids (*Plagioscion* sp./pescada) (Fig 6.20–24) and electric knife fish (Gymnotiformes).

### The fish spectrum

#### Synbranchidae

Swamp eels, also known as *muçum* or *anguilas de cuneta*, are decidedly the dominant group among all groups of fishes exploited at Loma Salvatierra and Loma Mendoza, a neighbor site contemporary with Loma Salvatierra [23,42]. Prior to this study, determination of swamp-eels to species level was constrained by the lack of a reference collection. Here, thanks to a referential of 150 individuals of modern synbranchids, we were able to document the presence of two species: *Synbranchus marmoratus* and *S*. *madeirae*. Identification was only possible for fragments of dentary and ectopterygoid bones [39]. Remains of the first of these species were much more common than those of the second: *S*. *marmoratus* accounted for about 17% of the total NISP (all phases included) whereas *S*. *madeirae* accounted for only about 1% (Table 1). Taking only the hand collected material, representation of swamp-eels is even greater, oscillating around 80%. Swamp-eels proportions were quite similar during phases 1 and 2 (the difference between the percentages of the two samples was not significant according to the t-statistic, 83 percent vs. 86 percent: z = -1.49, p < 0.12), gradually decreased during phases 3 and 4 and re-increased during phase 5 (data from phase 5 must be considered with care since total NISP is low compared to the other phases) (Fig 5).

The biology of *Synbranchus* eels is still poorly known. Individuals usually measure about 50 cm in length, but can reach up to 150 cm [43]. Size estimations of archaeological individuals revealed that larger individuals, varying from 60 to 90 cm, were selected (see section Size estimation). Synbranchids can breathe air, allowing them to thrive in very low-oxygen waters and to survive the dry season in buried channels in moist soil [44].

Mentions were made of *Synbranchus* consumption by Mojos indigenous groups among the Baures [45] and the Movimas [22] during the Jesuit occupation and later on among the Guarayos [14,46]. Today, swamp-eels do not appear to be an important element of commercial or subsistence fishing in Mojos [47].

#### Callichthyidae

The second most represented group was the armored catfishes. Three species were identified. From the largest to the smallest, these are *Hoplosternum littorale* (buchere, maximal length: 20 cm), *Megalechis picta* (simbao, maximal length: 15 cm) and *Corydoras* sp. (caracha, maximal length: 5 cm). Representation of Callichthyidae varied from 10 to 20% of the total NISP in phases 1, 2, 3 and 5, but much higher in phase 4, where there seems to have been an explosion of this group (Fig 5).

*Hoplosternum littorale* is undoubtedly the dominant species of this family in all five layers of occupation. Proportions of pelvic fins (Fig 6.29), fragments of the pectoral girdle (cleithrum, post-temporal, supra-cleithrum) were the anatomical parts best represented among the hand-collected material, while dermal plates were the anatomical part most represented in the sieved assemblage (dermal plates accounting for almost 50% of the NISP of sieved assemblages).

This family is characterized by the presence of bony armor composed of a series of bone plates (Fig 6.25). Since *Hoplosternum* is a facultative air-breather, the three species live in schools and can live in a broad range of aquatic environments, from oxygen-rich rivers to swampy areas where oxygen levels are extremely low [49]. In present-day Mojos, *Hoplosternum littorale* is concentrated in artificial lakes and ponds near Trinidad. Fish populations are even greater in stagnant waters than in permanent and rapid waters [50]. The “buchere soup” is considered to be a traditional meal in present-day Mojos.

Callichthyidae is one of the rare fish families displaying sexual dimorphism in anatomical characteristics: males generally have the pectoral fin slightly longer or stronger than females [51]. We have also noticed that in archaeological remains, surfaces of bones in male fins are porous, in contrast to those of females, in which the surfaces of bones are flat. According to Reis (1998) [51], the tips of pelvic fins of mature males twist in the form of a hook (Fig 6.29) during the reproductive period, which happens in present-day Llanos de Mojos, during the rainy season [48]. In the archaeological material, twisted fins correspond to about 3% of *Hoplosternum littorale* specimens (total N = 437). Thus, if environmental conditions have not drastically changed during the last 1000 years, our observations indicate that some *Hoplosternum* fishing could have taken place in the rainy season. This does not exclude the possibility that callichthyids could also have been caught during the dry season.

#### Lepidosirenidae

This family, the third most commonly recovered at Loma Salvatierra, is represented in South America by a single species, *Lepidosiren paradoxa* (lungfish). The lungfish has an elongated body (eel-like in form) and can reach a maximal length of 125 cm. As it possesses functional lungs, this species can survive under extremely dry conditions. Prearticular (Fig 6.3) and pterygoid bones are anatomical elements commonly recovered in assemblages. Like swamp-eels, lungfishes inhabit shallow and lentic waters and can survive from one high-water season to another buried in 50 cm deep galleries [48,52]. A progressive increase in the NISP of this taxon can be noted from phases 1 to 4, followed by an abrupt decline in phase 5 (Fig 3). Nowadays, lungfishes are not known to be consumed in Mojos.

#### Erythrinidae

Erythrinidae was the fourth most common family at Loma Salvatierra, where two species were recovered: *Hoplias malabaricus* (tiger fish, bentón or trahira) and *Hoplerythrinus unitaeniatus* (yayú), the former much more commonly than the latter (5% vs. 1% of the Total NISP, all phases included). Erythrinids possess conical and sharp teeth (Fig 6.5), elements that were commonly recovered in sieved material. The tiger fish (max length: 50 cm) (Fig 6.4–5) is widespread in South America and is commonly recovered in archaeological sites in freshwater contexts. The *yayu* is a smaller fish (max length: 330 mm) less widespread geographically than the tiger fish (Fig 6.6). Both species are highly adaptable to a range of environments, from free-flowing clear waters to shallow and lentic waters such as ponds and ditches [53]. Proportions of erythrinids varied between 4 to 11% along the five phases of occupation.

#### Serrasalmidae

Although quantitatively less important comparing to the taxa presented above, serrasalmids (pirañas or palometas) recovered at Loma Salvatierra are highly diverse. It was possible to distinguish two groups of dentaries: in the first, there are two rows of molariform teeth, a characteristic feature of herbivorous fishes such as *Mylossoma* (Fig 6.10), and in the second there is a single row (Fig 6.11) of triangular and sharp teeth (Fig 6.9), typical of carnivorous fishes [54]. Among this second group, we identified the presence of large-sized pirañas such as *Pygocentrus nattereri* (red-bellied piraña) and many other small to medium-sized pirañas such as those of the genera *Serrasalmus* and *Pristobrycon*. However, identification to species level was limited, given the large number of species existing in the region. Serrasalmids can inhabit ponds, lakes and edges of clear-water rivers. The first mentions of consumption of pirañas in the region are those of Eder 1985 [1772] [45] in the 18^th^ century. The Baures Indians used to fish them with hooks in sandy rivers. A half-century later, D'Orbigny (1843) [55] reported that the teeth of pirañas were used by Mojos Indians as scissors to cut their hair and weavers’ wires.

#### Cichlidae

The Cichlidae family, also known as *serepapas*, is large and diverse, reflected in Loma Salvatierra by a number of undetermined taxa. Three size classes of cichlids were recovered: small species such as *Acaronia* sp. (max length: 15 cm), *Cichlasoma* sp. (max length: 10cm); medium-sized species such as *Crenicichla* sp. (max length: 24 cm) and *Astronotus* sp. (max length: 24 cm); and large-sized species such as *Cichla* spp. (max length: 42 cm), the well-known *tucunaré* (Fig 6.22–24). All *serepapas* identified inhabit muddy bottoms of lentic waters except for the *tucunaré*, which is mostly found in lentic shores of clear-water rivers and lakes [48].

#### Loricariidae

The suckermouth catfishes (*zapato*, *carancho* or *bodo*) also constitute a very large family in terms of species richness and for that reason taxonomic identification was limited (Fig 6.17).

### *Synbranchus marmoratus* body size estimation

**Fig 7:**
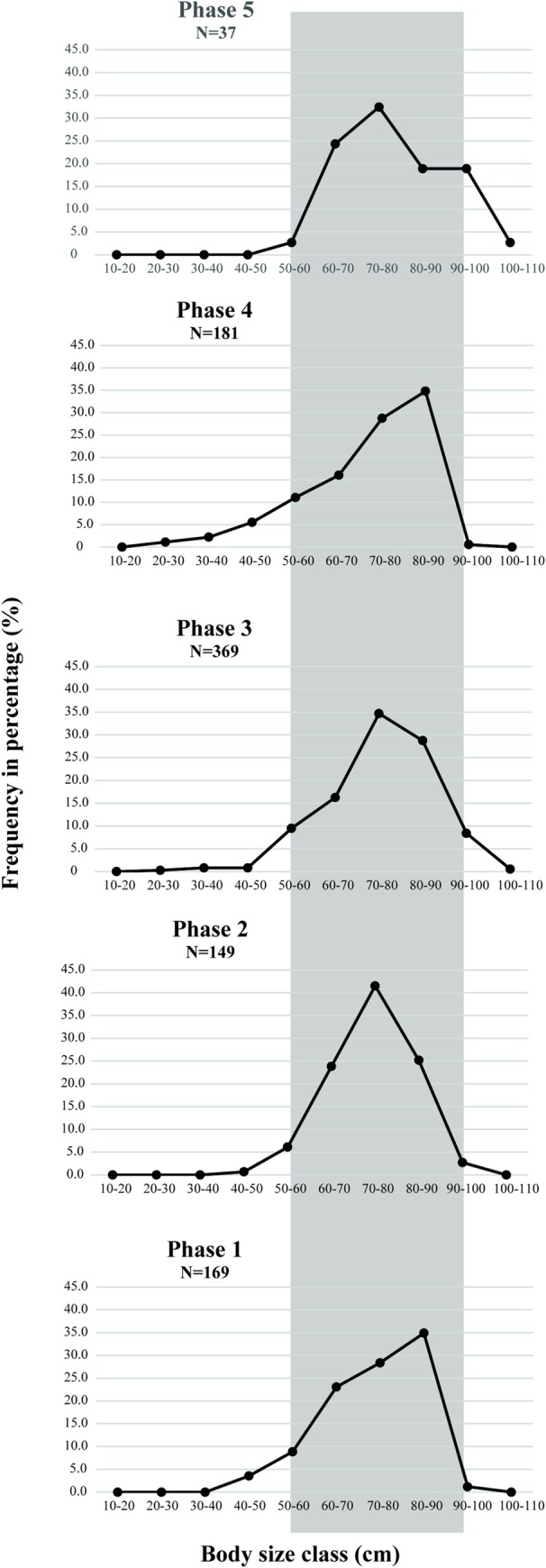
Frequency of estimated body size classes of archaeological swamp-eel *Synbranchus marmoratus* per phase of occupation.

The dentary (Fig 6.2) is the principal element recovered among the Synbranchidae assemblage and constitutes one of the only items that allow distinguishing between *Synbranchus marmoratus* and *Synbranchus madeirae* [39]. In order to estimate body size classes of archaeological populations, we measured the external body length of the dentary of *Synbranchus marmoratus*, the dominant species in Loma Salvatierra, and used the allometric model designed by [39]. We could apply the equation to 903 archaeological dentaries of *S*. *marmoratus*. Fig 7 shows that more than 90% of the archaeological individuals had estimated body sizes ranging from 50 to 90 cm, and about 40% had lengths between 70 and 80 cm. The largest archaeological specimen had an estimated size of 105 cm, which is 20 cm longer than the largest modern specimen of the reference collection, which measured 83 cm and weighed 530 g.

**Fig 8:**
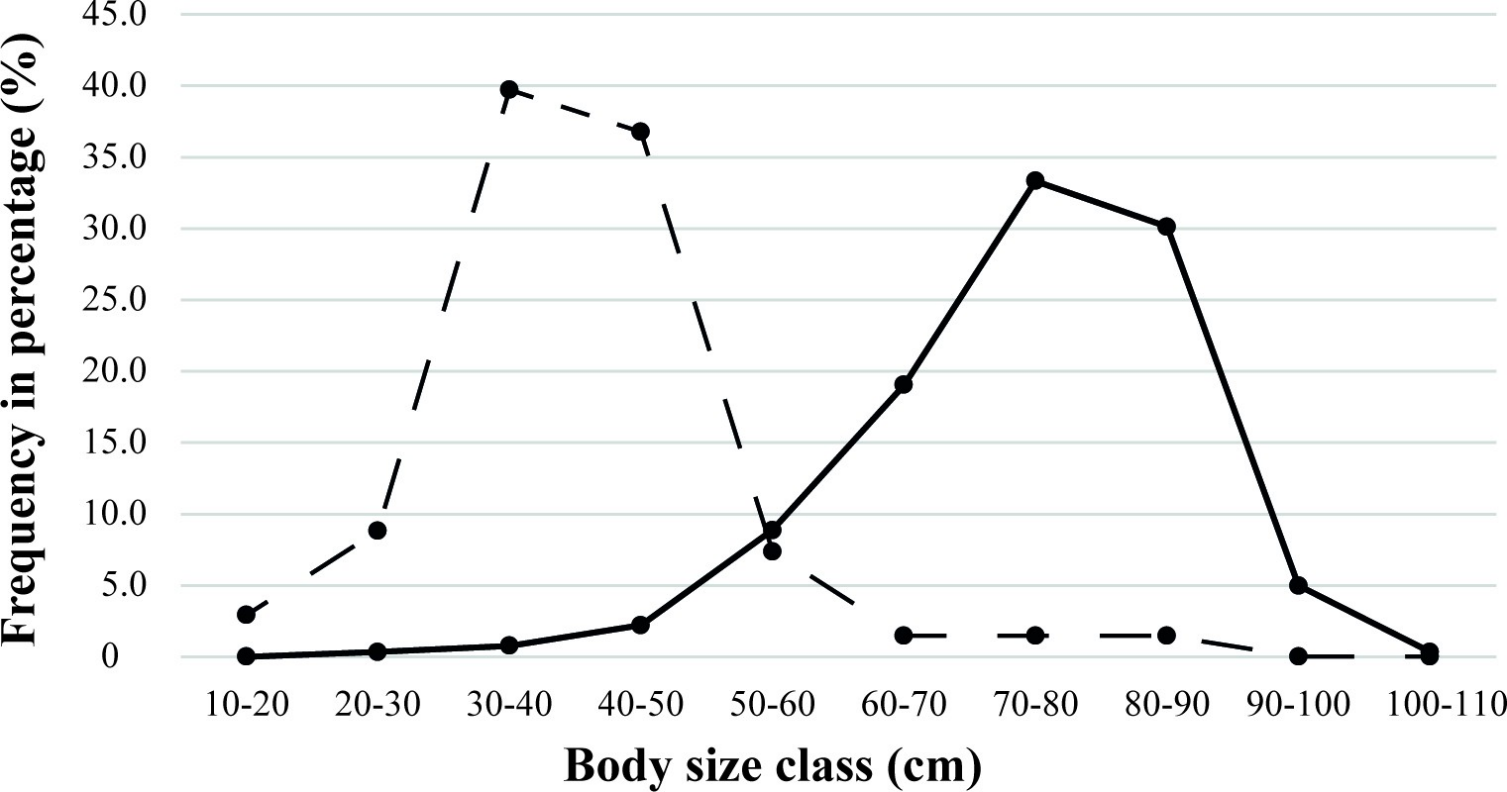
Swamp-eel body size comparison between archaeological (solid line) and modern reference collection (dashed line). For the archaeological assemblage (all phases confounded), N = 903 and for the modern reference collection, N = 68.

Comparing frequencies of body classes of archaeological assemblages (all layers combined) to the reference collection population (Fig 8), it is interesting to note that archaeological populations, measuring between 50 and 90 cm, are much larger than the modern ones. In the modern collection, most individuals measured between 30 and 60 cm.

From this information, we infer that there was selection of a specific body size class during the all five occupation phases of Loma Salvatierra, favoring large individuals. An alternative hypothesis is that mean size of individuals of this species was larger in ancient populations than in those of today. However, thus far there is no data to support this. Effects of overfishing can be ruled out, since fishing pressure on this species is currently very low. It seems, therefore, that size selection was undertaken by Loma Salvatierra fishers since larger individuals furnished more edible meat. We cannot exclude the possibility that ancient fishing techniques were more efficient than modern ones, or that the growth rhythm in ancient individuals was slightly different from that in present-day populations. These hypotheses should be tested in future research.

### Inferences about past-environments

In order to investigate past environments exploited as fishing spots, we mapped the main sources of water and of fish resources in the surroundings of Loma Salvatierra.

#### Nearby fishing spots: The specialization on swamp environments

It is important to remember that during the rainy season, the area of the site comprises extended aquatic territories, rich in fruits, seeds, and insects that attract a great number of fish. Fishes of many species spread over the savanna to reproduce, feed and seek refuge [56]. The rainy season is also a favorable period for the development of many macroinvertebrates such as gastropod mollusks of the genus *Pomacea*, recovered in large numbers from Loma Salvatierra. At the end of the wet period, most water and large numbers of fish become confined in smaller water bodies. With the beginning of the receding waters, the landscape can change rapidly, forming different environments:

*Swampy areas*: areas of shallow depressions where remaining waters are confined. They can occur throughout the blue area shown in the map of the Loma Salvatierra site (Fig 1).

*Curiches*: abandoned river cutoffs. The depressions left by the paleo-river that surrounded Loma Salvatierra before the period of human occupation certainly retained some water during the dry season.

*Artificial ponds (reservoirs)*: artificial circular ponds walled by piled earth, were constructed during the occupation of Loma Salvatierra (Figs 3 and 4). The function of the canals and ponds should be seen in a wider context of earthworks made to control water flow around Loma Salvatierra. The polygonal causeway, which encloses the site, blocked water from all sides. There are only three openings in the polygonal causeway that probably could have been closed by a sluice made of wood (see Fig 2). Water blocked at the Southwestern entrance of the paleo-river into the site would thus flow alongside the polygonal causeway to the Southeast and into the canal that starts at its southernmost point. This canal leads to pond 1 and, passing it, to pond 2 where it ends (Figs 1 and 2). A core sample from Pond 1 revealed that its original depth was about 1.8 m below the present-day surface. The layer of clay loam, rich in organic matter, that overlay hydromorphic (mottling) soil, was interpreted to be the bottom of the pond and produced calibrated radiocarbon dates between AD 1000 and AD 1200 [32]. Given diameters of approximately 30 m and original depths of 2 m, each pond could retain about 1410 m^3^ of water.

*Streams*: intermittent streams near Loma Salvatierra such as the Rio Cocharca and the Arroyo Mocovi (see Fig 1). These streams and their tributaries cross the savanna and dry up during the low-water season [24]. As many of the stream beds are now inactive or obstructed by sediments and plant debris so it is difficult to estimate their distance from the site, the level of water they contained, and the fish communities that inhabited them. It is clear, however, that the streams could have been a source of water at the beginning of the dry season.

**Fig 9:**
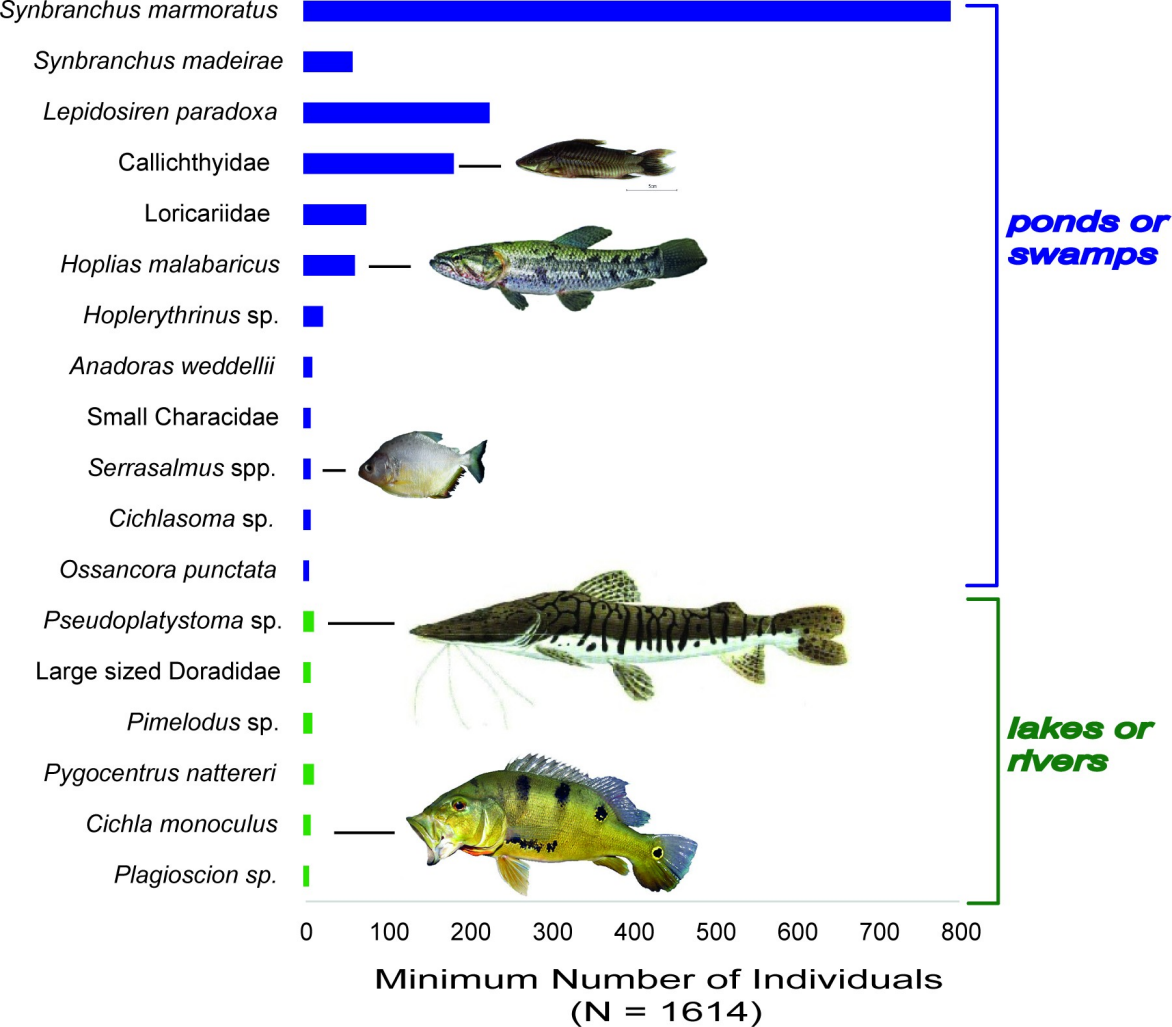
Habitats of main groups of fishes recovered at Loma Salvatierra (all phases combined).

*Canals*: excavated channels (about 1 meter deep), while not all mapped, irradiate from the site for at least 2 km [32]. According to these authors, these canals can connect villages to one another, to rivers, and to areas of cultivation. At Loma Salvatierra, Lombardo and Prümers (2010) suggest that, during the rainy season, the canals alongside the causeway participated in the drainage system and acted as a “funnel” directing water to the central pond [32]. During the dry season, these canals could also have retained stagnant water. In order to compare the aquatic environments and the fishes recovered at Loma Salvatierra, we classified the living habitats of main taxa based on existing studies that document fish communities present today in the area around Trinidad (Fig 9) [48,50,57].

Considering the dominant fish groups recovered at Loma Salvatierra, it is noteworthy that about 98% of the Minimal Number of Individuals is accounted for by fishes typical of shallow and lentic waters, suggesting that these seasonal environments were the most probable fishing spots exploited. The spectrum dominated by swamp-eels, armored catfishes, lungfishes and tiger fishes is very similar to the community recovered by Yunoki *et al*. (2013) [50] in present-day artificial ponds near Trinidad. Nowadays, these ponds’ fish fauna is constituted by 41 small and medium-sized species adapted to low levels of dissolved oxygen.

#### Exogenous taxa

The remaining 2% of the MNI correspond to larger-sized fishes, such as *Pseudoplatystoma* sp., *Cichla* sp., *Oxydoras* sp. and *Pterodoras* sp. that infrequently inhabit the small streams, ponds, or stagnant waters that surround Loma Salvatierra and, as such, can be considered exogenous species. The rivers closest to Loma Salvatierra are the Rio Ibare, situated about 35 km distant, and the Rio Mamoré, about 55 km from the archaeological site. All exogenous taxa are found in the rivers. Also, about 7 km to the South of Loma Salvatierra there are two perennial savanna lakes, the Laguna San José and the Laguna Azul (Fig 1). These lakes are shallow (1–3 m) and long (Laguna San José is about 5 km long and 2 km wide) [28]. Fed by local precipitation, their surface area and water level vary considerably over the year. In a study carried out by Pouilly and Rodríguez (2004) [28] in the similar savanna lakes of Coitarama and Laguna Suarez, both tiger-striped catfish (*Pseudoplatystoma* sp.), and tucunaré (*Cichla* sp.) were captured. The low number of individuals of these taxa recovered at Loma Salvatierra shows that the perennial water sources could have been sporadically used as fishing areas. Furthermore, it is not impossible that occasionally, some large-sized fishes could have spread into flooded areas during the high-water season.

Despite the similar taxonomic spectra of the two excavation units, these uncommon taxa (sting-rays, large catfishes, large cichlids, sciaenids and electric knife fish) were only present in EU 4, which corroborates Jaimes Betancourt´s (2012) [27] hypothesis that it was an area where festivities could have taken place.

## Discussion

The existence of of large human settlements in interfluvial areas of the Llanos de Mojos has been known since the first Jesuits entered the region in the 17^th^ century: “*There seems to be more than four thousand souls in this Province of Moxos*, *scattered far away from each other in more than fifty villages*, *some along the streams*, *others along the lakes*, *others in the countryside over the pampas*, *and all independent from each other (…)”*,our translation [58]. We observe that the position of Loma Salvatierra at the border of a savanna area subject to inundation could have been a strategic choice in terms of water management. Retention of annual rainfall through the presence of both natural depressions and a paleochannel along with a system of articulated canals and ponds at Loma Salvatierra could have produced water retention allowing people to inhabit this environment through the dry months. Indeed, the potential of aquatic fauna of South American swamps and savannas, in terms of biomass and richness of food resources, is an idea that many ecologists, such as Junk *et al*. (1989) and Pouilly *et al*. (2004), [56,57] have documented but is still little explored by archaeologists.

Debates in archaeology concerning the protein availability have overlooked these resources and the environments of the savanna [6,7,9,59,60]. At Loma Salvatierra the construction of canals and ponds is dated to at least 1000–1200 AD and as such may their function likely was enhancing upon the way natural features were observed to function. The landscape transformations from other areas of the Americas serve as a reference to better understand how aquatic systems were managed.

### American examples of fishing in built structures

The Chinampas network of the valley of Mexico is one of the first examples of hydraulic management found in the Americas. Chinampas were garden islands dated to as old as 2000 years ago and constructed by the piling of earth in shallow lakes [61–63]. The artificial islands (about 100 m long by 5–10 m wide) were arranged in a grid-like system separated by canals, where fishes were netted or speared. According to Beveridge and Little (2000) [64], the Chinampas constituted “an integrated wetland agriculture/aquaculture system”. The hydraulic management of swamps through the construction of ditches has also been documented in regions of the Central American lowlands such as Campeche and Quintana Roo and in different periods dating from the last 2000 years until European Conquest. Canals were built cross-cutting river floodplains and sometimes associated with ridged fields, indicating that canals may have served as multi-functional structures, for transportation but also serving as fish refuges [65,66].

There are at least two South American examples of the exploitation of fish in built structures. Both are situated in savanna areas located on the periphery of the Amazon region and both began flourishing around 500 AD. One example is the pond-corral system described on Marajo Island, in the Amazon estuary. Roosevelt (1991) [67] and Schaan (2008) [68] discovered river-linked lakes, dams, and excavated ponds associated with archaeological sites noted for their platform mounds dating back to 500 AD. Despite the probable fishing activities in the built waterways, very little information is available on which fish groups would have inhabited these environments. Preliminary zooarchaological data presented by Roosevelt (1991) [67] for one of these platforms (Teso de los Bichos) indicates that the great majority of the faunal assemblage is composed of small-sized fish, such as armored catfish (*Hoplosternum littorale*), tiger fish (*Hoplias malabaricus*), piranhas (*Serrasalmus* sp.), and indeterminate, small-sized characins. Roosevelt interprets the pond system as strategic water and fish storage for the dry season.

The second example comes from the Venezuelan Llanos. This region is a large savanna situated on the Amazon’s northern periphery, between the Sarupa and Apure rivers. A complex system of ridged fields and canals was found at the site of La Calzada de Paez, occupied from 500 to 1400 AD. According to Zucchi (1984) [69], built canals and the areas excavated while building causeways and ridged fields formed a water retention network that created favorable environments for the development of aquatic fauna. Fishes and other aquatic animals constituted a non-negligible resource exploited in this Pre-Hispanic system [69]. In La Calzada de Paez, a zooarchaeological study revealed that the dominant fish taxa were *Hoplias malabaricus*, *Pimelodus* cf. *ornatus*, and *Hoplosternum* sp. [4], a spectrum very similar to that of Loma Salvatierra.

The aquatic management systems from the Americas were built upon an underlying knowledge of the relationship between the annual rainfall regime and topographic features to create fishing environments. The functioning of built fishery systems has been explored experimentally. In San Antonio, Albion Island (Belize) raised fields were reconstructed on a low plain and the canals, thus excavated, were subsequently surveyed for fish. The most common were swamp-eels (*Synbranchus marmoratus*) and small *Cichlasoma* spp. [65]. The frequent appearance of swamp-eel has also been described in modern and active irrigation-ditch systems in Honduras [70]. The potential association of swamp-eels with built environments is noteworthy, in light of the over-representation of *S*. *marmoratus* at Loma Salvatierra. Even though swamp-eels are no longer harvested in present-day Llanos de Mojos [47], they are commonly found in ponds and urban, earthen roadside gutters, and during the dry season they may even be found buried under 1 m of mud [71].

### The potential contribution of fish ponds to the Loma Salvatierra fishery system

The presence of ponds and canals has been mentioned for Pre-Hispanic settlements of the Llanos de Mojos, however the fish species that occurred in these structures have not been explored. These data are now available for the region of Loma Salvatierra. To explore the relationship of fish with managed structures we have evaluated the types of constructions in other parts of the Llanos de Mojos. We suggest that at least two configurations of ponds can be observed in Mojos. In the first configuration, a few ponds, usually no more than three per settlement with some associated canals, were dug along the margins of forest islands or in the immediate surroundings of an occupation [72]. This configuration seems to occur in the northwestern Mojos near Santa Ana del Yacuma (see Fig 13 in Lombardo 2010) [73]; in the southwestern Mojos near the Apere river (see Fig 6 in Erickson 2000) [74], near the Maniqui river (see Fig 1 in Dickau *et al*. 2016) [75], and near San Ignacio de Mojos (Erickson 1980) [76]; and in the southeastern Mojos near Trinidad (Langstroth Plotkin 1996) [77] and San Javier (Erickson and Balée 2006) [78]. The second configuration of ponds occurs in the northeastern Mojos near Baures and has been documented by Erickson (2000) [8], McKey *et al*. (2016) [20] and Blatrix *et al*. [21]. In this case, ponds (generally varying between 0.5-2m deep and 10-30m in diameter) occur in larger numbers in a systematic association with V-shaped structures that cross savanna plains and have been explained as long earthen weirs [20]. These ponds have been interpreted as a source of aquatic resources and drinking water during the dry season [8]. No information exists about the fish consumed at the associated archaeological sites.

The layout of Loma Salvatierra´s ponds corresponds to the examples given for the first configuration. Similar to in Ibibate Mound Complex, the ponds are circular pits, 0.5 to 2 m deep, dug in the immediate surroundings of the mound [78]. It may be possible to relate the fish spectrum from the Loma Salvatierra site with the ecological characteristics of the habitats currently occupied by these species and the potential for ponds and/or canals to be one of the habitats. Despite the taxonomic richness (35 genera identified) in the archaeologically recovered fish remains, four taxa are dominant: swamp-eels (*Synbranchus* spp.), armored catfishes (*Hoplosternum* spp.), lungfish (*Lepidosiren paradoxa*), and tiger-fish (*Hoplias malabaricus*). While swamp-eels have a wide distribution, the characteristic assemblage of actual temporary ponds in the Amazon basin includes the families of all of the archaeologically dominant fish (the families Erythrinidae, Callichthyidae and Synbranchidae) [79]. Ponds present unstable environmental conditions (low levels of oxygen and fluctuating water levels) in their lentic and stagnant waters. These fish families present adaptations to this habitat such as the synchronization of their life cycles with the seasonal dynamics of pond´s filling and shrinking and evolved respiratory mechanisms including aerial breathing [49,80,81]. The correspondence of the actual assemblage in ponds and archaeological fish spectrum provides support for the idea that the ponds at Loma Salvatierra or analogous environments, including the paleochannel, were fished by the Pre-Hispanic inhabitants.

The size of fish species recovered in archaeological assemblages can also suggest particular ecological characteristics of the habitats. Lowe-McConnell (1964) [82] showed that deep water bodies are needed for the growth of larger fish (e.g. large catfishes, cichlids and sciaenids), while ponds are more typically inhabited by small fish. In this regard, the fishing of small and shallow bodies of water may be associated with an assemblage of small fish such that found at Loma Salvatierra. The archaeological sampling using fine mesh recovered quantity of very small fishes such as 20 g *Corydoras* sp., 30 g cichlids, 70 g callichthyids and 50 g erythrinids and other small-sized fish, even if an exact quantification is difficult the distribution of taxa in water screened samples may provide a more realistic spectrum. Small and shallow water bodies are encountered in the immediate vicinity of Loma Salvatierra, specifically in the constructed ponds and paleochannel where area and depth are limited.

At Loma Salvatierra the prevalence of small sized fish, the species composition of families common to temporary ponds, and the association of built ponds with the last phase of occupation are three convergent lines of reasoning that support the hypothesis of the use of managed structures in fishing activities. The canals that encircle Loma Salvatierra have not yet been associated with agricultural areas or practices, but most canals in the Monumental Mounds Region were drainage canals that probably improved the rate of run off and the utility of the savanna for agriculture [38]. The integration of ponds into these earthwork systems has not yet been associated with agricultural practices and need to be examined in reference to both drainage and other functionalities.

Many authors have claimed that artificial pond structures are a first step in a “fish domestication” process [83, 84]. According to Teletchea and Fontaine (2014) [83], fish domestication is a long and ongoing process of development and adaptation that combines captivity with human control of fish life cycles. These authors propose “levels” of domestication starting with stocking trials and acclimatization to captivity. The last level is achieved when the full cycle of reproduction occurs in captivity independent of wild inputs. The difference between domesticated and non-domesticated fish is that reared populations experience major changes in morphology (e.g. in body and shape) and behavior [84].

Pond-like structures have been mentioned in fish rearing [84]. One of the best known cases is the domestication of the common carp (*Cyprinus carpio*), initiated by the Romans in pond-rearing structures at the beginning of the first century BC. Carp were captured in the Danube and stored in reservoirs, but managed carp were being produced in pond systems including spawning and growing ponds. Holding fish in systems such as earthen ponds and cages can be considered as imposing “unintentional artificial selection” [84] or as a type of “proto-aquaculture” [85]. As postulated by Beveridge and Little (2006) “Many proto-aquaculture activities relied on some sort of holding facility. The simplest to construct would have been earth ponds. In some parts of the world these would have been little more than mud walls constructed to temporarily hold water and fish following the seasonal flooding of a river” [64]. Furthermore, according to Harache (2002) [86], it is not surprising that the first fish species to be domesticated were freshwater fish, which are easily separated from rivers and naturally reproduce in ponds.

Although we wish to avoid falling prey to labels (“proto”, “pre”, “post”), it seems that Loma Salvatierra´s ponds present some functional similarities with the structures cited above: during the rainy season fish fauna is naturally spread in the savannas [50,87], and at the beginning of the dry season water recedes and fish could be stored, an idea already proposed for the Baures region by Erickson (2000) [8]. However, it is important to remember that applying modern concepts of “fish domestication” and “aquaculture” to ancient patterns is a delicate issue, especially where “there is still a transition from capture to culture of aquatic animals, sometimes making it difficult to determine when capture ends and culture begins” [83].

The presence of the ponds and canals is clear evidence of intentional transformation and management of the landscape, mainly because pond systems need regular maintenance to remain operational: labor is required to avoid natural and/or anthropogenic sedimentation. Fish holding seems to be one of the functions of the ponds, but not the only one as this type of network was beneficial for drainage and water holding on a seasonal basis. In other words, Loma Salvatierra inhabitants probably recognized the potential benefits of holding fish and water but we cannot affirm that either was the original purpose for the pond’s construction. Meanwhile, the water system was open to external environments and fish mobility was not restricted during times of inundation. Therefore landscape transformation and management probably left no defined modification on the biological structure of the fish populations inhabiting the ponds, as would be expected under true domestication.

## Conclusions

The annual inundations of the Amazonian savannas create extensive seasonal aquatic ecosystems and environments that are highly productive [56,88] and far from being hostile to human settlement or deficient in animal protein. The fish spectrum reveals that a high diversity of fishes, species from at least 35 genera, were exploited and consumed. This constitutes the richest fish spectrum so far documented for the Llanos de Mojos region of Bolivia. Swamp eels (*Synbranchus* spp.) are over-represented in the spectrum, which may be explained by the ability of this species to adapt to extreme dry conditions. This resource would not have been limited to the rainy season, and thus the greater quantities may be due to the use of this secure resource during long periods of drought. The dry season availability of armored catfishes (*Hoplosternum* spp.), lungfish (*Lepidosiren paradoxa*), and tiger-fish (*Hoplias malabaricus*) in small water bodies would also provide resources for populations living year-round in the interfluvial areas of the Mojos. The paucity of larger-bodied fish that inhabit larger water bodies, such as the closest permanent water source, a lake seven kilometers from the site, suggests an infrequent contribution of exogenous fish. The savanna, in contrast to the large Amazonian rivers, presents a distinct set of fishing habitats where humans likely established specific fishing strategies.

The Loma Salvatierra archaeological site contains a mosaic of built landscape transformations, both intentional, mounds, canals, causeways, and ponds, and unintentional as in the areas excavated during the construction of the earthworks. The complex network of natural and artificial depressions seems to have captured surplus seasonal rainfall and retained water during the dry season, demonstrating that these groups knowledge of the rainfall regime and topographic features influenced the construction of managed aquatic landscapes. The ecological characteristics of the paleochannel and pond network would have been analogous to modern canals that have been found to be amenable niches for the archaeologically dominant fish taxa, swamp eels (S*ynbranchus marmoratus*),lungfish (*Lepidosiren paradoxa)*, armored catfish (*Hoplosternum* sp.), and tiger-fish (*Hoplias malabaricus*). These taxa and other small sized fish typical of lentic and shallow waters, recovered in the archaeological site support the hypothesis that waters in local depressions were used for fishing activities. The ponds and canals built during the last phase of occupation probably served multiple purposes including water storage and drainage, but fish management is one of the possibilities. It is likely that pre-Hispanic hydraulic management systems in the Llanos de Mojos were comprised of multi-component and multi-functional structures, as already emphasized by Denevan (1966) [15], Erickson and Balée (2006) [78] and McKey *et al*. (2014) [89]. The best-known aquatic management systems in the Americas integrated areas of agriculture and fishing, however this is not apparent at Loma Salvatierra where the cultivated spaces are not yet evident.

The chronological fluctuations in fish taxa abundance lead us to look for the possible relationships between changes in the fishing activities and both fluctuating precipitation regimes and human investments in managing the landscape. During which periods did the fish population change? As managed landscapes probably evolved parallel to natural changes over time, this question is currently impossible to answer. But there are new directions that may assist in future investigations, specifically how the inundated zones changed over time, the chronology of pond construction and remodeling, inclusion of incidental water retention features in mapping of the hydraulic networks, fine grained records of precipitation, and investigation of fish age profiles for the multiple species that grow in pond environments.

A further question is how to reconstruct evidence of potential fish storage and holding activities. Would such practices have left signatures on bones of stored fish communities? Which of the artificial structures would have been used for this activity, and how would they have been used?

The results from Loma Salvatierra suggest that pond systems across the neotropical lowlands need to be considered as potential loci for fishing activities and that these practices can be separated from fishing in open waters on the basis of the zooarchaeological record. Ponds occur in association with other earthworks in at least three other regions of pre-Hispanic settlement on different savannas from around 400 to 1400 AD: the Bolivian Llanos de Mojos, the Venezuelan Llanos, and the Marajo Island. The potential of aquatic resources (fish, aquatic mammals, and invertebrates) in earthwork systems has been hypothesized by Zucchi (1984), Roosevelt (1991), Erickson (2000) [67,69,74] and others, and confirmed in this study. The importance of pond systems in regional economies can be explored by the study of faunal remains and greater analysis of their hydraulic functioning. The similarities in the built landscape across the lowlands cannot be discounted despite the kilometers that separate these savannas. Additionally, ponds have been documented at other Amazonian archaeological settlements, including the regions of Santarém [90,91] and Manaus [92], indicating that an investment in water and aquatic animals management may be a basin wide phenomenon that is less understood in upland and *várzea* environments. Combined zoorchaeological, archaeobotanical, and geoarchaeological approaches should provide insights for building a macro-regional understanding of water management systems and fishing practices.

## Supporting information

S1 Table Distribution of fish remains (NISP) from the Loma Salvatierra archaeological site (Bolivia) samples collected by hand and by water screening (2mm and 1 mm meshes) across all excavations. NI = No information; * Minimum volume.
(DOCX)
