# Supplementary material for: Pre-Hispanic fishing practices in interfluvial Amazonia: Zooarchaeological evidence from managed landscapes on the Llanos de Mojos savanna
Source: PLoS One. 2019 May 15;14(5):e0214638. doi: 10.1371/journal.pone.0214638 (PMC6519797; doi:10.1371/journal.pone.0214638)
Supplement: S1 Table — NI = No information; * Minimum volume. (DOCX) [file pone.0214638.s001.docx]

|  | **Phase 1** | | **Phase 2** | | **Phase 3** | | **Phase 4** | | **Phase 5** | |  | **Total (all phases)** | |
| --- | --- | --- | --- | --- | --- | --- | --- | --- | --- | --- | --- | --- | --- |
|  | **Hand collection** | **water screening** | **Hand collection** | **water screening** | **Hand collection** | **water screening** | **Hand collection** | **water screening** | **Hand collection** | **water screening** |  | **Hand collection** | **water screening** |
| **Sample Volume (l)** | NI | 17 | NI | 1.5 | NI | 51 | NI | 10 | NI | 10 |  | NI | 89.5* |
| Callichthyidae | 73 | 128 | 23 | 17 | 326 | 179 | 818 | 832 | 22 | 250 |  | 1262 | 1406 |
| Cichlidae | 1 | 29 | 0 | 0 | 20 | 10 | 15 | 242 | 0 | 25 |  | 36 | 306 |
| Erythrinidae | 17 | 86 | 7 | 5 | 103 | 72 | 72 | 568 | 7 | 22 |  | 206 | 753 |
| Lepidosirenidae | 34 | 5 | 31 | 15 | 200 | 18 | 127 | 171 | 58 | 16 |  | 450 | 225 |
| Loricariidae | 6 | 1 | 27 | 16 | 40 | 16 | 82 | 78 | 8 | 2 |  | 163 | 113 |
| Doradidae | 5 | 2 | 2 | 1 | 2 | 0 | 2 | 0 | 0 | 0 |  | 11 | 3 |
| Indet Characiformes | 1 | 2 | 1 | 1 | 2 | 1 | 8 | 24 | 1 | 5 |  | 13 | 33 |
| Siluriformes | 2 | 15 | 2 | 1 | 24 | 3 | 1 | 23 | 4 | 2 |  | 33 | 44 |
| Synbranchidae | 711 | 147 | 612 | 107 | 1777 | 137 | 1043 | 998 | 249 | 65 |  | 4392 | 1454 |
|  | **850** | **415** | **705** | **163** | **2494** | **436** | **2168** | **2936** | **349** | **387** |  | **6566** | **4337** |
